# Anatomy, digital radiography and cone-beam computed tomography of Western European hedgehog *(Erinaceus europaeus)* skull

**DOI:** 10.1186/s12917-024-04280-9

**Published:** 2024-09-20

**Authors:** Jakub Jędrzej Ruszkowski, Maciej Zdun, Marcin Bartłomiej Arciszewski

**Affiliations:** 1 Department of Animal Anatomy, Poznan University of Life Sciences, Wojska Polskiego 71C, 60-625 Poznan, Poland; 2 Department of Animal Anatomy and Histology, University of Life Sciences in Lublin, Akademicka 12, 20-950 Lublin, Poland

**Keywords:** Imaging, Insectivora, Osteology, Radiology, Wildlife

## Abstract

**Background:**

European hedgehogs *(Erinaceus europaeus)* are small insectivorous mammals common in Europe. With increased urbanization, the species become more frequent in the cities and near other human dwellings. The anatomy of the species with the use of diagnostic imaging techniques along with the classical anatomical methodology has not been researched before. In addition to the description of this species' skull, the data may contribute to progress in hedgehog medicine, delivering a basis for diagnosing and treating head trauma in this species.

**Results:**

Cadavers of 30 European hedgehogs have been used to study the anatomy of the head of the species. Along with classical anatomical techniques – latex and corrosion cast specimens, x-ray, and cone-bean computed tomography scans were performed. The methods were then compared, and the detailed anatomy of the head was described. The skull of the Western European hedgehog was elongated and oval in shape, and could be divided into two distinct regions: one formed of neurocranial bones and the other of facial bones. The neurocranium was composed of the following bones: the occipital, interparietal, parietal, frontal, temporal, sphenoid, ethmoid, and pterygoid bones. The following facial bones were identified: the incisive, nasal, maxilla, zygomatic, palatine, vomer, and the mandible. The most important findings include the primitive tympanic bullae, the absence of a supraorbital foramen, and the lacrimal bones, which were indistinguishably fused with the maxillae.

**Conclusions:**

The results of the study may be used in future research on the comparative anatomy of the other members of the Eulipotyphla order. With the increase of hedgehogs in the cities, it is important to establish new diagnostic and treatment protocols for wildlife rehabilitation centers. Anatomical and radiological descriptions may be used as a base for such work. The anatomical features of the hedgehog skull described in the study may prove useful for further studies from a comparative anatomical perspective. Additionally, the data may serve as a basis for developing identification guidelines for archaeological studies and forensic research.

## Background

Hedgehogs are small, nocturnal, insectivorous animals living in Europe, Africa and continental Asia. There are 17 species of hedgehogs belonging to five genera: Erinaceus, Paraechinus, Mesechinus, Atelerix and Hemiechinus. European hedgehogs *(Erinaceus europaeus)* are distributed across Europe from Iberia through Italy and up to Scandinavia [1, 2]. The most common habitats for hedgehogs are deciduous forests, grassland, and rural areas, but they are becoming more frequent in urban areas. Human dwellings provide them with high food availability, the absence of predators and many hiding places [2, 3]. One of the biggest threats for hedgehogs living in urban areas is communication traffic – due to the higher number of hedgehogs in the cities, they are increasingly becoming traffic victims [4, 5]. With an increasing number of injured hedgehogs, more animals are transported to Wildlife Rehabilitation Centres (WRC) [6]. It implies the necessity of acquiring knowledge about species-specific veterinary care protocols.

The trauma of unknown origin and road accidents, including bone fractures and head trauma, are among the most frequent injuries leading to the death of hedgehogs in rehabilitation centers [7, 8]. Among the noted fractures, the axial skeleton – spine, and skull are common. With the increasing number of WRCs worldwide, it is essential to understand and classify these animals’ most frequent health issues. The basis of clinical knowledge of a different kind of bone trauma is the anatomy of the skeleton. The increase of such trauma in wildlife creates the need to establish detailed anatomical descriptions followed by treatment protocols for species that have not been studied before and may be challenging for veterinary clinicians.

The skeleton of different species of hedgehog has been described before a few times, but the articles describe mostly appendicular skeleton [9–11] and one describes axial skeleton in general [12]. This article aimed to describe the anatomy of the skull in detail using macroanatomy, radiography, and cone-bean computed tomography (CBCT) techniques. In addition to the description of this species' skull, the data may contribute to progress in hedgehog medicine, delivering a basis for diagnosing and treating head trauma in this species.

Using radiological examination and other advanced imaging techniques (e.g., computed tomography) as an additional source of information along with manual preparation for the anatomical study is becoming more popular in anatomical science [13–15].

Hashemi used radiological examination to describe the appendicular skeleton of the Southern white-breasted hedgehog *(Erinaceus concolor)* [10].

The radiological features of the anatomy of the head of European hedgehog have not been studied in detail in the past. Among all species of hedgehogs, only Long-eared hedgehogs *(Hemiechinus auritus)* skull anatomy has been described using both skeletal specimens and radiography [16].

Since the European hedgehog is a protected species in some European countries, more and more attention is paid to active actions to improve the quality of life of this species. Many wild mammal rehabilitation centers are being established, and more and more veterinarians specialize in treating wild animals. The progress of this branch of veterinary medicine forces the creation of diagnostic and treatment protocols, thanks to which it can be developed. The description of anatomical structures and imaging tests can be an excellent basis for developing procedures in imaging diagnostics or hedgehog surgery.

Radiological studies have also been used in forensic veterinary sciences to assess the cause of death or as evidentiary material in cases of animal abuse [17]. Since European hedgehogs are protected by law in Poland and many other countries, this study may also contribute to this topic as a reference.

The use of 3D reconstruction in veterinary anatomy is becoming a new standard for teaching at universities [18, 19]. A digitalized model of the skull or other bones is accessible and can be used worldwide e.g. in countries that cannot get a specific species’ bone material. This is of more importance in protected species, since the cadavers may be hard to obtain.

## Methods

### Animals

All procedures done to accomplish the goal of the study were approved and carried out in accordance with the appropriate regulations and permits (Regional Directorate for Environmental Protection in Poznan (Poland): WPN-II.6401.366.2020.TE) due to the fact that hedgehogs are protected by Polish law (Regulation of the Minister of the Environment of 16 December 2016), on the protection of animal species (Journal of Laws, item 2183) and (Journal of Laws 2020, item 26).

Cadavers of 30 European hedgehogs admitted to a wildlife rehabilitation center in Poznan, Poland were used in the study. The cause of death was different than trauma to the head. For each consecutive data were obtained: species, sex, and body weight. 15 adult males and 15 adult females have been used in this study. Animals with body weight above 500g were included in the study.

### X-ray and CBCT scans

The radiological part of the study was performed at the University Centre for Veterinary Medicine in Poznan, Poland. Dorsoventral and lateral radiographs of the heads were performed using a digital radiography system (Examion Maxivet DR, Fellbach, Germany) with parameters of 50kV and 2 mAs.

Cone-bean computed tomography scans (Fidex Animage, California, USA) of 3 males and 3 females were performed with scanning parameters of 110 kVp, 0.08 mAs pet shot, 20.48 mAs (Total mAs). The scans were post-produced in FidexGUI (version 3.6.0, Animage, USA) to obtain the 3D reconstruction of the skulls.

### Anatomical preparation

Specimens were skinned, and soft tissues were cut out and immersed into a detergent solution (Persil). The temperature used for this process was 40 °C. The process lasted 10 days. After the maceration process, any remaining soft tissue was manually removed under running tap water. Bones were left to dry for 10 days.

To visualize internal bones, 5 skulls were gently defragmented with anatomical forceps in the sutures.

After the drying process, bones were photographed using a digital camera (Canon 650D) and archived. The photographs were saved in JPEG format. GIMP v2.10.18 digital image editing software was used to process the photographs and create figures.

The anatomical terminology used was based on the 6th edition of the *Nomina Anatomica Veterinaria* (NAV), where its applicable to domestic mammalian species [20]. In the case of species-specific structures, the terminology was based on Butler’s work [21].

## Results

### Skull as a whole *(Cranium)*

In a lateral view, the hedgehog skull has an oval shape, elongated and tapering rostrally. The ventral surface of the skull is straight. The superior surface of the skull is slightly convex in its’ parietal portion. The zygomatic arch consists of three parts: the zygomatic process of the maxilla, the tiny zygomatic bone, and the zygomatic process of the temporal bone. The most rostral part is broad and directed caudo-ventrally. Further, the arch becomes slender and directed caudo-superiorly. The orbit has an oval shape and is delimited rostrally by the maxilla laterally by the zygomatic arch. Superiorly and caudally, the orbit opens freely into the temporal fossa. There is no zygomatic process of the frontal bone, or any other remains of the postorbital bar. The orbital regions’ bones have numerous openings: ethmoid foramen, optic canal, orbitorotundum foramen and rostral alar foramen leading to the alar canal, that ends with caudal alar foramen.

The mandible is well-developed with visibly shaped processes: coronoid, condylar and angular. On the lateral surface there is deep masseteric fossa. Two mental foramina ends the mandibular canal.

The adult hedgehog of both sexes has 36 teeth. The dental formula is I 3/2, C 1/1, P 3/2, M 3/3.

The use of three techniques allowed for the creation of a complete image allowing for a detailed description of the anatomy of the hedgehog's skull. Anatomical preparation requires a lot of time and the use of precise techniques and tools. This method is also susceptible to human errors and often requires a significant amount of material. We decided to use radiological and tomographic examinations because these are common imaging techniques used in veterinary clinics. X-ray examination is a common method and available in many places, but it has some limitations. Due to the two-dimensional nature of X-ray images, frequent overlapping of anatomical structures does not allow for a detailed assessment of individual details. CT examination, however, allows for obtaining a more detailed image with the possibility of creating a 3D reconstruction. Therefore, it allows for the assessment of bone material in a multidimensional manner.

#### Neurocranium

**Fig. 1:**
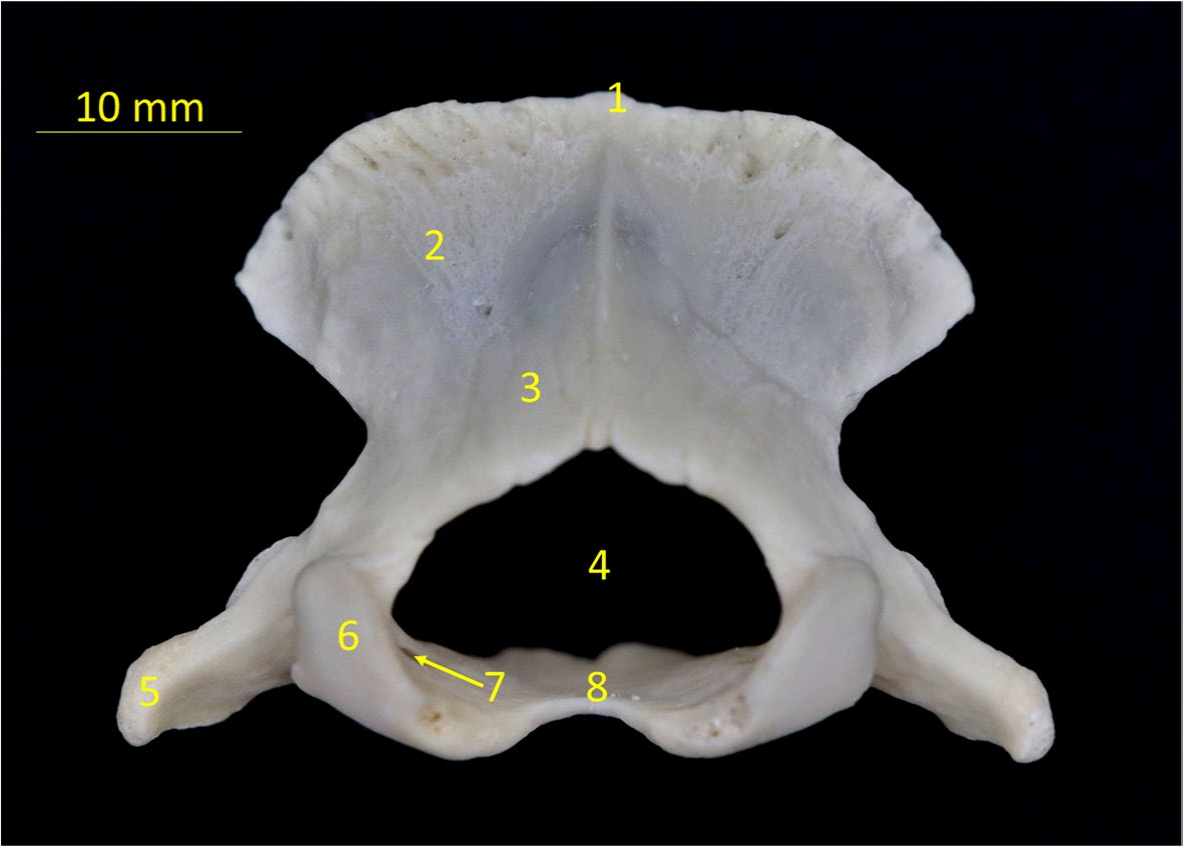
Isolated specimen of the occipital bone of the European hedgehog. Caudal view. 1 – nuchal crest, 2 – squamous part, 3 – lateral part, 4 – foramen magnum, 5 – paracondylar process, 6 – occipital condyle, 7 – hypoglossal canal, 8 – basilar part

Occipital bone *(Os occipitale)* (Fig. 1).

The occipital bone builds up the caudal wall of the skull. It is an unpaired bone that forms an atlantooccipital joint that links the skull to the vertebral column. It is built of the squamous part *(squama occipitalis)*, the basilar part *(pars basillaris)*, and two lateral parts *(pars laterales)* (Fig. 1). On the superior edge, it forms a highly prominent nuchal crest *(crista nuchae)*. The crest divides ventrally into two parts. The rostral one terminates on the mastoid process *(processus mastoideus)* connected with a post-glenoid process *(processus retroarticularis)*, and the caudal one at the paracondylar process *(processus paracondylaris)*. On the back of the squamous part, where it links to both lateral parts there is occipital external protuberance *(protuberantia occipitalis externa)*, which extends ventrally. Underneath it, there is a foramen magnum *(foramen magnum)*. It is built by lateral parts of the occipital bone, and is not reaching the squamous part superiorly. Ventrally the occipital bone forms two occipital condyles *(condylus occipitalis)*. The condylar foramina *(foramen condylaris)* are absent. The ventral condylar fossa *(fossa condylaris ventralis)* is wide and it separates the occipital condyle from the paracondylar process, which links to the temporal bone *(os temporale)*. The paracondylar processes do not exceed the line of the condylar processes ventrally. The ventral condylar fossa is perforated by the hypoglossal canal which lies near the wider jugular foramen.

The basillar part of the occipital bone with sphenoid bones *(os sphenoidale)* forms the bottom of the skull cavity *(cavum cranii).*

This bone could be visualized in both ventro-dorsal (VD) and lateral (L) projection on x ray and in the 3D reconstruction from a CT scan. The bone outline, general shape of the squama, condyles, and processes were visible on both examinations. On the 3D reconstruction, additionally, the shape of the foramen magnum and nuchal crest were visible.

**Fig. 2:**
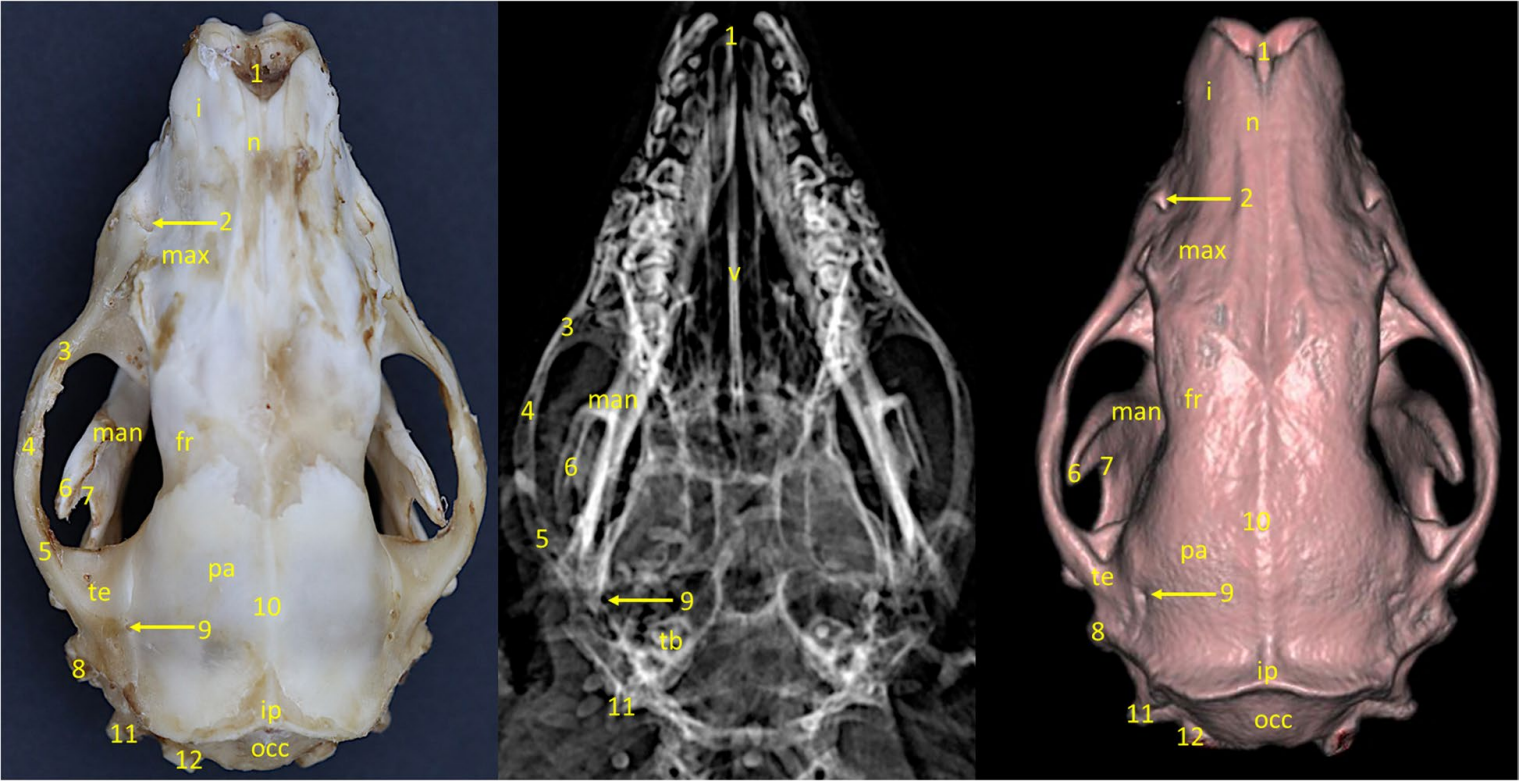
Dorso-ventral view of the European hedgehog skull. From the left: a specimen, an x-ray, a 3D reconstruction from CBCT scan. n – nasal bone, i – incisive bone, max – maxilla, fr – frontal bone, man – mandible, pa – parietal bone, te – temporal bone, ip – interparietal bone, occ – occipital bone, 1 – nares, 2 – infraorbital foramen, 3 – zygomatic process of the maxilla, 4 – zygomatic bone, 5 – zygomatic process of the temporal bone, 6 – coronoid process, 7 – sigmoid notch, 8 – mastoid process, 9 – temporal foramen, 10 – external sagittal crest, 11 – paracondylar process, 12 – occipital condyle

Interparietal bone* (os interparietale)* (Fig. 2.)

The interparietal bone lies between the parietal and occipital bones. It is elongated and lies along the nuchal crest. The interparietal bone could not be distinguished from surrounding structures on both radiographic pictures and 3D reconstruction.

**Fig. 3:**
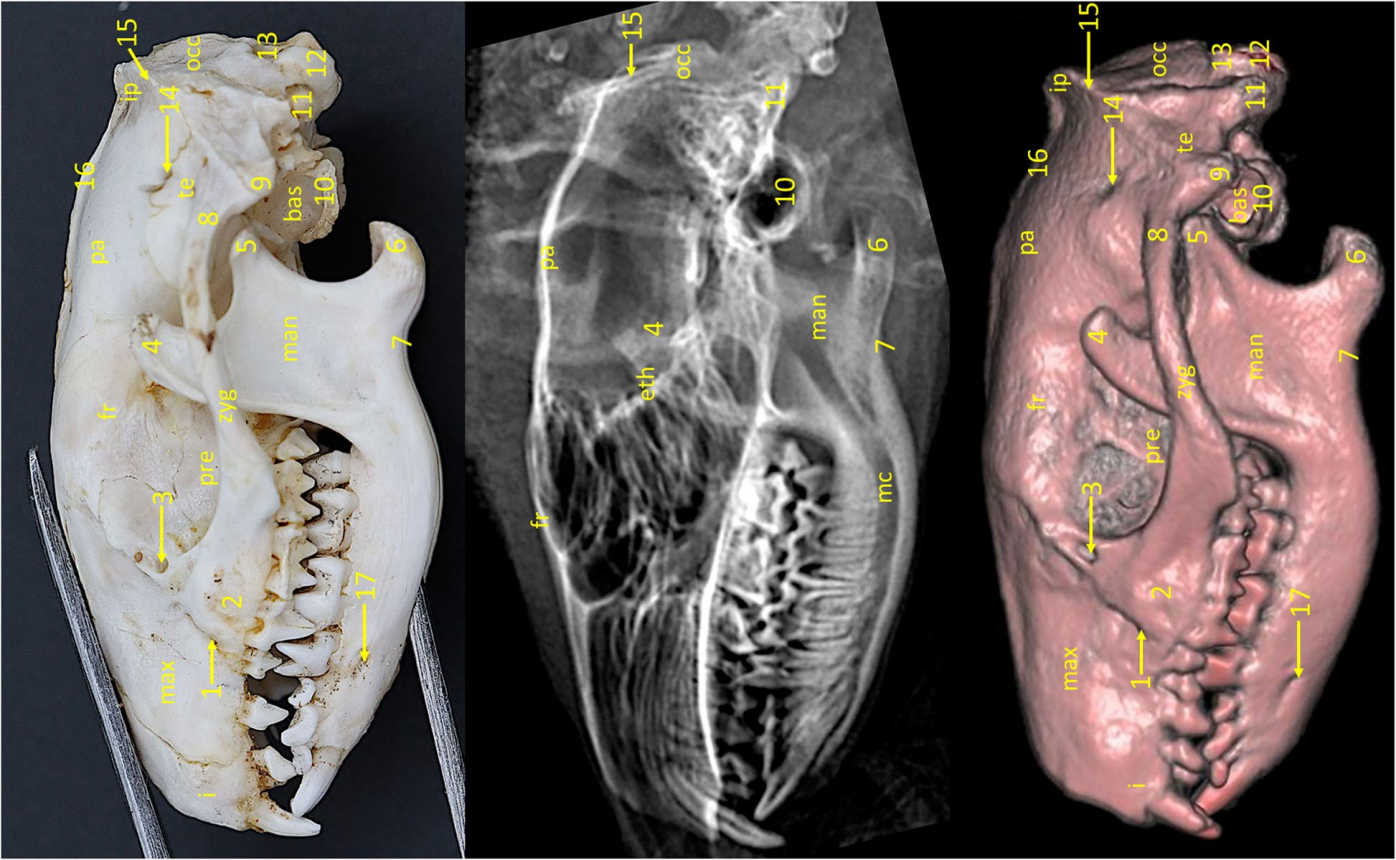
Lateral view of the European hedgehog skull. From the left: a specimen, an x-ray, a 3D reconstruction from CBCT scan. i – incisive bone, max – maxilla, fr – frontal bone, pre – presphenoid bone, zyg – zygomatic bone, man—mandible, pa – parietal bone, te – temporal bone, bas – basisphenoid bone, occ – occipital bone, eth – ethmoid bone, mc – mandibular canal, 1 – infraorbital foramen, 2 – zygomatic process of the maxilla, 3 – lacrimal foramen, 4 – coronoid process, 5 – condylar process, 6 – angular process, 7 – vascular impression, 8 – zygomatic process of the temporal bone, 9 – mastoid process, 10 – tympanic process, 11 – paracondylar process, 12 – occipital condyle, 13 – foramen magnum, 14 – temporal foramen, 15 – nuchal crest, 16 – external sagittal crest, 17 – mental foramen

Parietal bone* (os parietale)* (Figs. 2 and  3).

Parietal bone builds up the superior part of the cranial cavity. It connects to the frontal and sphenoid bones by the frontal border *(margo frontalis)*, temporal bone by the saggital border *(margo saggitalis)*, and interparieral bone by interparietal border *(margo interpartietalis)*. Axially, where both parietal bones connect there is the easily visible sagittal external crest *(crista saggitalis externa)*. The shape of this bone conditions the anatomy of the temporal fossa *(fossa temporalis)*. The outline of the bone has been visible on both radiographic and 3D reconstruction images (Fig. 2).

Frontal bone* (os frontale)* (Figs. 2 and 3).

Frontal bones are located at the central part of the skull. It builds up a superior part of the orbit *(orbita)* in the form of a moderately prominent frontal sinus *(sinus frontalis)*. It articulates rostrally with the nasal *(os nasale)* and maxillary bones *(maxilla)*, ventrally with maxillary and sphenoid bones and caudally with parietal bones and sphenoid bone (Fig. 3). It does reach the temporal bones caudoventrally in a short section. Together with the wing of the presphenoid bone, it builds up a medial wall of the orbit and delimits it superiorly. At the superior part of the bone, where two frontals connect, there is a crest that prolongs between the rostral edge of the parietal bones and connects to the sagittal external crest. The crest divides between the orbits into to crests descending to the lacrimal region. The supraorbital foramina *(foramen supraorbitale)* are not present in this species.

The exact shape of this bone could not be identified on an x-ray image, due to overlapping with other skull structures. It could be visible on the 3D reconstruction.

**Fig. 4:**
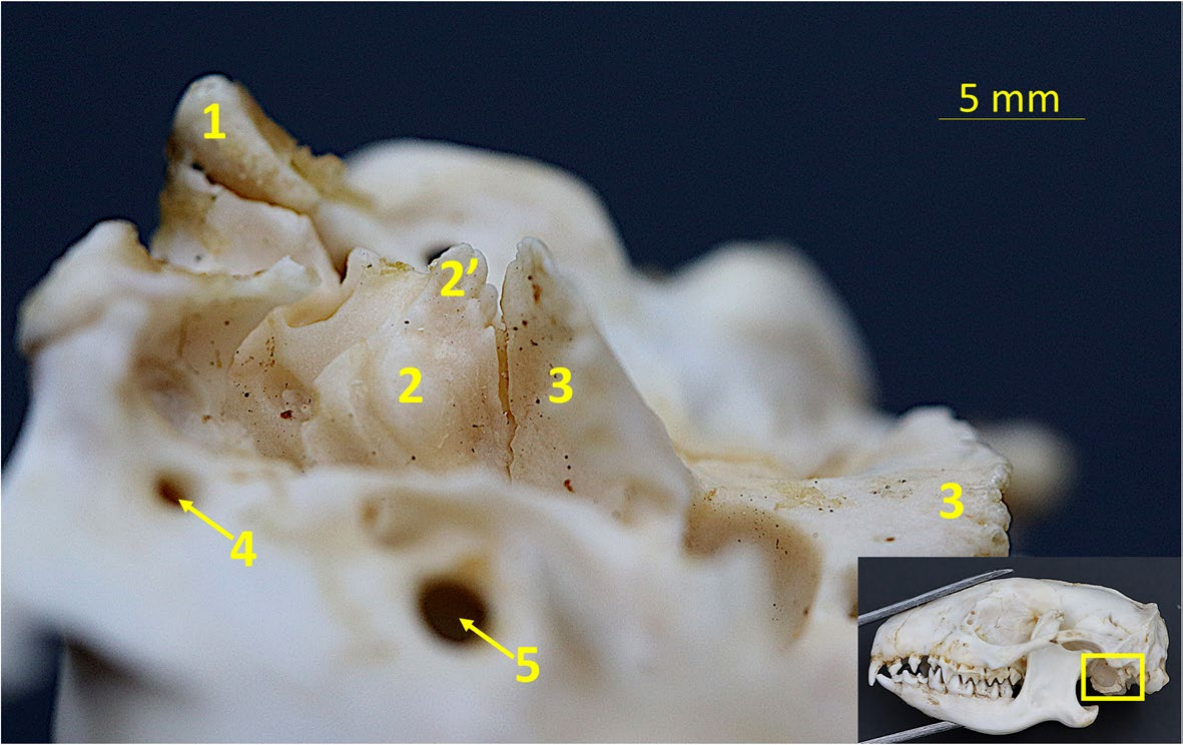
Ventral view of the skull of the Western European hedgehog. The close-up photograph, taken laterally to the tympanic bulla, presents an image of the interior of the tympanic bulla region. It is notable that the tympanic bulla has not been subjected to mechanical opening, as the ectotympanic has been removed. 1 – paracondylar process, 2 – petrous part of the temporal bone, 2’ – tympanic process, 3 – tympanic wings, 4 – retroarticular foramen, 5 – oval foramen

**Fig. 5:**
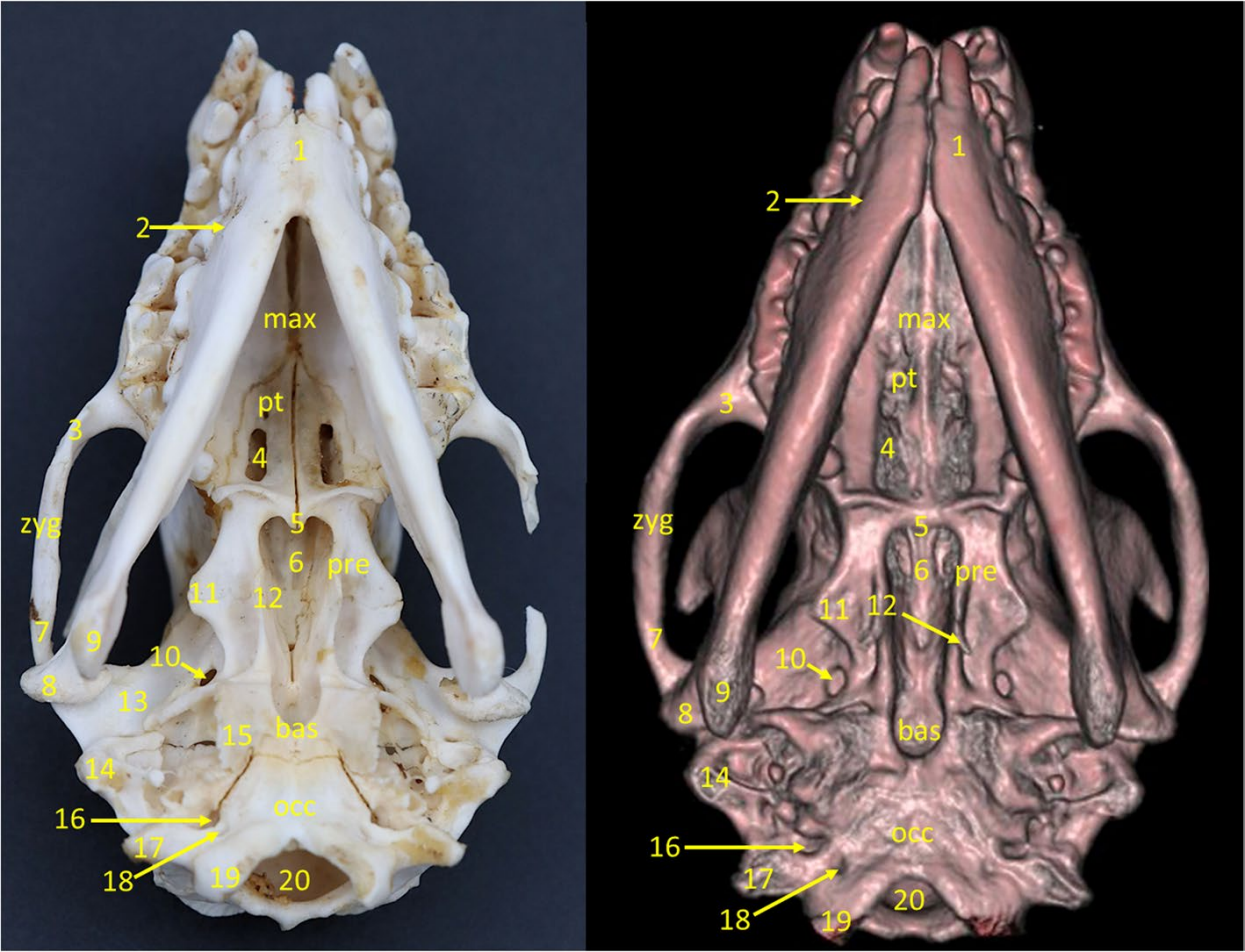
Ventro-dorsal view of the European hedgehog skull. From the left: a specimen, an x-ray, a 3D reconstruction from CBCT scan. max – palatine process of the maxilla, pt – horizontal lamina of the palatine bone, zyg – zygomatic bone, pre – presphenoid bone, bas – basisphenoid bone, occ – occipital bone, 1 – mandibular symphysis, 2 – mental foramen, zygomatic process of the maxilla, 4 – major palatine foramen, 5 – caudal nasal spine, 6 – choanae, 7 – zygomatic process of the temporal bone, 8 – condylar process, 9 – angular process, 10 – oval foramen, 11 – horizontal lamina of the presphenoid, 12 – pterygoid hamulus, 13 – mandibular fossa, 14 – mastoid process, 15 – tympanic process, 16 – jugular foramen, 17 – paracondylar process, 18 – hypoglossal canal, 19 – occipital condyle, 20 – foramen magnum

Temporal bone* (os temporale)* (Figs. 3, 4 and 5).

The temporal bone connects caudally to the occipital bone and medially to the presphenoid *(os presphenoidale)* and basisphenoid bone *(os presphenoidale)*. Two parts of this bone have been identified in European hedgehogs—the petrosal *(pars petrosa)*, and squamous part *(pars squamosa)*. The most lateral part of the temporal bone is its’ squamous part. Rostrally, it forms the long zygomatic process of the temporal bone *(processus zygomaticus ossis temporalis)*. On the superior side, it is performed by one or more opening-temporal foramen *(foramen temporalis)* leading to the temporal canal *(canalis temporalis)*. On the underside of the bone, there is a moderately flat mandibular fossa *(fossa mandibularis)* with retroarticular foramen *(foramen retroarticulare)*, and a slightly prominent retroarticular process *(processus retroarticularis)*. This process links to the mastoid process—a round-shaped process arising for the petrous part of the temporal bone. Behind the basal part of the process lies the petrous part of the temporal bone. The petrous part has the tympanic process *(processus tympanicus)* that, along with the tympanic wing of the basisphenoid forms caudolaterally the lesser, the bony part of the tympanic bulla *(bulla tympanica)* (Fig. 4). The tympanic bone forms and arched plate not ossified with the rest to the skull—ectotympanic *(tympanum)*. The ectotympanic limits the cavity-like space, though it cannot be called a tympanic cavity *(cavum tympanicum)*, since the whole structure is not ossified. This space is closed from the medial aspect by a tympanic process of the basisphenoid.

The temporal bone was visible in both radiographic and 3D reconstruction images. On the VD radiograph the shape and size of the zygomatic process of the temporal bone could be seen. On the lateral radiograph, tympanic bulla and cavity can be distinguished. The shape of the bulla and the glenoid fossa could also be distinguished.

Sphenoid bone* (os sphenoidale)* (Figs. 3, 4 and 5).

The sphenoid bone is not described in animal anatomy as a standalone structure. Instead, two smaller bones are distinguished in its place: the presphenoid (os presphenoidale) and the basisphenoid (os basisphenoidale) (Fig. 5). However, for the sake of a more comprehensive description and a more nuanced understanding of their structure, the two parts are usually treated as a single entity. The body *(corpus)* and wings *(alae)* can be distinguished in both. Both parts are connected by the intersphenoidal synchondrosis *(synchondrosis intersphenoidale)*. The sphenoid bone connects to the occipital bone by the sphenooccipital synchondrosis* (synchondrosis sphenooccipitale)*.

The presphenoid rostrally joins from the medial side to the pterygoid hamuli *(hamulus pterygoideus)*. It also connects with pterygoid bone with hollow horizontal lamina *(lamina horizontalis)* forming pterygoid fossa *(fossa pterygoidea)*.

**Fig. 6:**
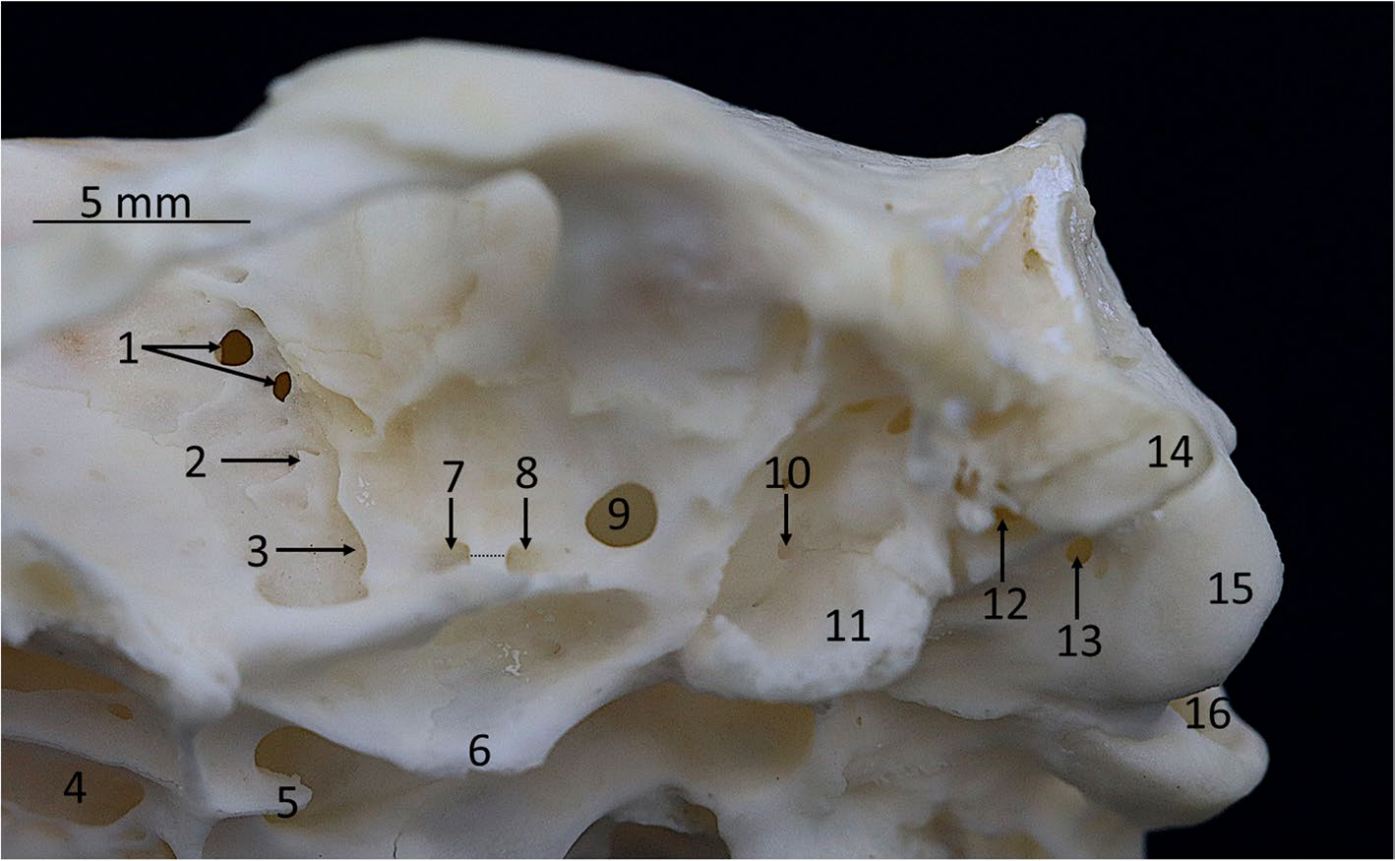
Ventrolateral view of the European hedgehog skull. 1 – ethmoid foramina, 2 – optic foramen, 3 – orbitorotundum foramen, 4 – major palatine foramen, 5 – caudal nasal spine, 6 – horizontal lamina of the presphenoid, 7 – rostral alar foramen, 8 – caudal alar foramen, 7–8 – alar canal, 9 – oval foramen, 10 – carotid foramen, 11 – tympanic process, 12 – jugular foramen, 13 – hypoglossal canal, 14 – paracondylar process, 15 – occipital condyle, 16 – foramen magnum

It is divided into the body *(corpus)* and wings *(alae)*. Those structures limit the rostral cranial fossa *(fossa cranialis rostralis).* Near the casual end of the presphenoid body there is a sulcus chiasmatis *(sulcus chiasmaticus)* that leads to optic canals. Laterally and caudally, there is a deep sulcus that lies on the wings of both pre- and basisphenoid leading do the orbitorotundum foramina (Fig. 6). Above the sulcus chiasmatis lies the ethamoid foramen, that opens in the side of the ethmoid fossa *(fossa ethmoidale)*.

The basisphenoid lies caudally to the presphenoid, is also divided into the body and wings, and its body forms paired, expanded, semicircular bony lamina, called auditory process that protrude caudolaterally. To this process, attaches the tympanic bone, which is not ossified with the rest to the skull. This plate is not fused with the crescenic lamina arising from the basisphenoid. Those processes form partially the wall of the primitive tympanic cavity, which has a small opening in the superior part—the carotid foramen *(foramen caroticus).* Between those structures, there is a deep, round-shaped recess. The bone wall creating this recess is not perforated.

On the superior part of the body there is a slight depression—hypophysialis fossa *(fossa hypophysialis)* and a slightly prominently crest caudally—dorsum sellae *(dorsum sellae).* Laterally to this fossa lies the groove for the ophthalmic and maxillary nerves *(sulcus nervi optici et maxillaris)*. It reaches the ortbitorotundum foramen.

The wings of the basisphenoid entirely surround the most visible foramen of this area—an oval foramen *(foramen ovale)*.

The sphenoid bone could be visible on both VD radiographic and 3D reconstruction images. The general shape of the bone,the recess of the basisphenoid, and the oval foramen were visible. The intersphenoid and sphenooccipital synchondrosis were better visible on the radiographic image.

Ethmoid bone *(os ethmoidale)* (Fig. 3).

The ethmoid bone is located at the border between the viscerocranium and the neurocranium between the orbital walls. It forms the rostral border of the cranial cavity, in the form of a perforated lamina, and divides it from the nasal cavity. It is built of four parts: a cribriform plate *(lamina cribrosa)*, a perpendicular plate *(lamina perpendicularis)*, an orbital plate *(lamina orbitalis)* and an ethmoid labyrinth *(labyrinthus ethmoidalis)*. The cribriform plate is divided into two ethmoid fossae *(fossa ethmoidalis)* perforated by ethmoid foramina *(foramen ethmoidale)* that differ in size. Between the two triangular fossae, there is a crista galli *(crista galli)* that is prominent in the superior part and is hidden between the domed parts of the cribriform plate ventrally.

Rostrally, from the ethmoid labyrinth, the endo- and ectoturbinates arise.

Due to the intracranial location of the ethmoid bone, only endo-and ectoturbinates were visible on the lateral radiographic image. On the 3D reconstruction image, main parts of the ethmoid bone were visible after removing the frontal and parietal bones.

Pterygoid bone *(os pterygoideus)* (Fig. 5).

The pterygoid bone lies between the palatine bone and the sphenoid bone. Ventrally it forms a hamular process *(hamulus pterygoideus)*, which links to the presphenoid bone. Laterally the pterygoid bone has hollow horizontal lamina that, together with a similar structure to presphenoid bones forms a pterygoid fossa *(fossa pterygoideum)*. Above the lamina occurs the pterygoid foramen *(foramen pterygoideum)* that opens the pterygoid canal *(canalis pterygoideus)*. The shape of the bone was visualized in the 3D reconstruction image. Due to the arched shape of the bone, radiographic images are not good for assessing its shape and structure.

### Viscerocranium

Incisive bone* (os incisivum)* (Figs. 2 and 3).

Incisive bones are the most rostrally protruding bilateral bones of the cranium. They are divided by the nasal bones superiorly and articulate with maxilla caudally. It consists of the body *(corpus),* alveolar process* (processus alveolaris)* and nasal process *(processus nasalis).* The nasal process has the biggest surface of all parts. On the ventral side incisive bone has three alveoli *(alveolus)* for upper incisors. They are moved laterally from the medial plane of the skull. There are small palatal processes of the incisive bone *(processus palatinus ossis incisivi)* limiting a palatine fissure *(fissura palatina)*. These fissures are semilunar in shape. The lateral part of the external surface bone is slightly convex with a little thickening on the rostral edge. Superiorly, above the medial interincisive region, lies the vomer bone. The bones are visible on both lateral radiographic and 3D reconstruction images. The 3D reconstruction allows the assessment of the shape of the rostral edge of the bones in detail.

Nasal Bone* (os nasale)* (Figs. 2 and 3).

The nasal bones are paired and projected over the nasal cavity between incisive and maxillar bones. The external surface of the bone is convex and the internal—is concave. Between the rostral tips of paired nasals, there is a slightly marked nasal notch created by bilateral nasal processes *(processus nasalis)*. On the ventral side, the ethmoidal crest *(crista ethmoidalis)* protrudes on the caudal part of the bone. The bones are visible on both lateral radiographic and 3D reconstruction images.

Maxilla *(maxilla)* (Figs. 2 and 3).

Maxillae are wide, strong, paired bones building a major part of the facial bones. It has a very wide palatal* (processus palatinus maxillae)* process, which builds a large surface of the hard palate ranging from incisive to molar teeth. Rostral part of this bone—the body *(corpus maxillae) contacts the incisive bone, superior part contacts nasal and frontal bone. *On the internal surface, the conchal crest (crista conchalis) protrudes to link with the central. nasal concha. Maxilla, with its zygomatic process *(processus zygomaticus maxillae)* creates a rostral part of The zygomatic arch *(arcus zygomaticus)* composed of two other bones. Slightly eminent facial tuberosity *(tuber faciale)* is penetrated by the infraorbital canal *(canalis infraorbitalis)*, which begins as maxillary foramen *(foramen maxillare)* located in a bone groove inside the orbit, in the pterygopalatine fossa, and ends with infraorbital foramen *(foramen infraorbitale)* at the. rostral part of the facial tuberosity. It also forms alveolar processes *(processus alveolaris)* for maxillary teeth. 

The lacrimal bone is not distinguishable from the maxilla. Part of the maxilla, located in a topography of the lacrimal bones in other species, is located at the rostral edge of the orbit and protrudes rostrally as a lacrimal process penetrated by a lacrimal foramen *(foramen lacrimale) *situated on the edge of the superior margin of the orbital rim (margo supraorbitalis). It opens above the infraorbital foramen, on the external part of the orbit. Due to the overlapping of anatomical structures of the head, the lacrimal bone cannot be distinguished on the radiographic image. The 3D reconstruction image allows to assess the shape of the bone and it’s anatomical structures—the lacrimal process and foramen.

Radiography allows us to visualize only the general shape of the maxilla. On the 3D reconstruction, both shape and anatomical structures like its’ zygomatic process, palatal process and infraorbital foramen can be assessed.

Zygomatic bone* (os zygomaticumv)* (Figs. 2, 3 and 5).

The zygomatic bone is a tiny, oblong bone building up the connection of the zygomatic process of the maxilla and the zygomatic process of the temporal bone from the lateral side. The shape of the zygomatic bones is visible on both radiographic and 3D reconstruction image.

*Palatine* bone* (os palatinum)* (Fig. 2).

The palatine bone is visible on the ventral surface of the skull and has two parts: horizontal *(lamina horizontalis ossis palatini)* and perpendicular plates *(lamina perpendicularis ossis palatini)*. A horizontal plate of the palatine bone lies between the caudal edges of the palatal processes of the maxilla. It has one, narrow major palatal foramina divided in most specimens by the thin bony plate into two, and single or double round-shaped minor palatal foramina caudally. From the caudal edge, also called a free edge *(margo liber)* of the palatine bone, protrudes an unpaired caudal nasal spine *(spinum nasale)*. On the external of this plate, the transverse crest protrudes reaching the last molar teeth alveolus. The perpendicular plate articulates with maxillary and ethmoid bones. The part of the plate creating an orbital wall—the orbital process *(processus orbitalis)* has a small surface. Overlapping of many structures on both VD and lateral projection of the radiographic image didn't allow assessing the palatine bones' details. On the 3D reconstruction image, the general shape and majority of the details can be assessed.

*Vomer (vomer)* (Fig. 2).

The vomer is an unpaired bone that lies on the ventral part of the nasal cavity *(cavum nasale)*, and it is the ventral part of the nasal septum *(septum nasale)*. It is connected to nasal crests *(crista nasalis).* The vomer is visible on the radiographic image, but due to its intracranial location, only its rostral part is visible on the 3D reconstruction image.

**Fig. 7:**
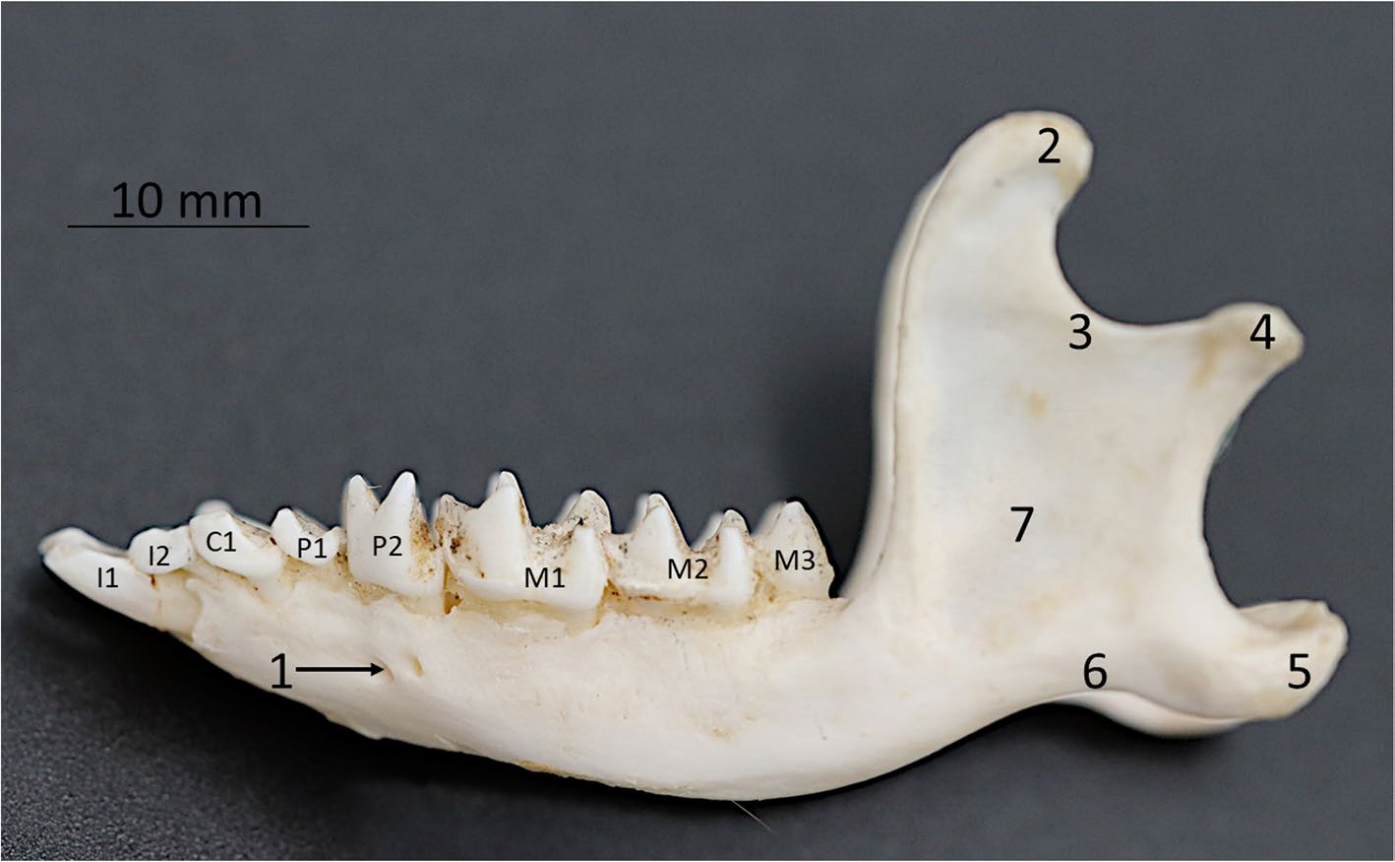
Lateral view of the European hedgehog mandible specimen. 1 – mental foramen, 2 – coronoid process, 3- sigmoid notch, 4 – condylar process, 5 – angular process, 6 – vascular impression, 7 – masseteric fossa

*Mandible (mandibula) (*Fig. 7*)*

The mandible has two main parts: the body *(corpus mandibulae)* and the ramus *(ramus mandibulae)*. Both parts unite in the angle of the mandible *(angulus mandibulae).*

The mandibular ramus *(ramus mandibulae)* is well-developed, triangle-shaped with the deftly visible coronoid *(processus coronoideus)*, condylar *(processus condylaris)* and angular processes *(processus angularis)* (Fig. 7). The coronoid process is significantly higher than a condylar process with a deep sigmoid notch (*incisura mandibulae)*. The condylar process is well-developed. It is transversely broadened with an oval-shaped articular face. The angular process is hooked and slightly bent to the medial direction. On the medial side, it has a prominent horizontal crest. On lateral surface of the mandibular ramus there is a deep masseteric fossa *(fossa masseterica)*. On the medial surface of the mandibular ramus, there is a pterygoid fossa *(fossa pterygoidea).* Ventrally from this fossa, there is a single mandibular foramen *(foramen mandibulae)* and a prominent mylohyoid line *(linea mylohyoidea)*. The body of the mandible is elongated and narrow and has alveolar processes for 8 teeth. Two mental foramina *(foramen mentale)* are located at a height of first mandibular premolar. Mandibular symphysis is narrow and reaches first two premolar teeth.

The lateral radiographic projection allows the assessment of the body of the mandible with the angular process and a coronoid process. However, it is impossible to differentiate sides due to the overlapping of both the left and right mandible. On the 3D reconstruction image, the whole shape mandible was clearly visible, along with its openings.

## Discussion

The skull of the European hedgehog has some characteristics that allow to identify it as an insectivore mammal's skull—slender, elongated skull and mandible and longer snout [22]. The description of the osteological characteristics of the skull coincides with the results of Miller's work (1920) [23]. The general shape of the skull and its variability has been measured before in studies concerning dietary habits [22] or evolutionary differences between different species of hedgehogs [24, 25]. Anatomical landmarks used in such studies are often made on prominent or characteristic anatomical structures. Detailed anatomical descriptions of a species can help identify such landmarks in future studies from the fields of comparative or evolutionary anatomy. Anatomical characteristics of the general shape of the skull have been similar to those described before by Butler (1948) [21]—the skull is relatively broad and low with a broad palate, the zygomatic arch is elevated towards the posterior, and the upper profile of the skull rises to the highest point above the orbit. The described skulls had relatively prominent crests on sagittal and lambdoidal sutures, and the braincase was flat and broad. Those characteristics were described previously as typical for adult individuals in other species of Eulipotyphla [26].

The shape of the maxilla and the caudal range of the fronto-maxillary sutures were similar to those described by Thomas (1918) [27].

The naso-maxillary suture clearly separated the incisive and frontal bones from the maxilla. Such feature was described as typical for the European population of *Erinaceus concolor* in contrast to the Near and Middle East population, where the suture was significantly shorter. Based on this measurement, Krystufek (2002) determined two skull morphotypes—*roumanicus and concolor.* According to this division, the skull of adult *Erinaceus europaeus* belongs to the *roumanicus* morphotype [24].

The shape and anatomical features of basicranium, such as the appearance of the supramental fossa and foramina of this region, are similar to those described in *Erinaceus amuriensis* [28].

A few of the osteological features found in the Western European hedgehog in this study were also described in other species before. The nasal notch has been describe before in dog [29]. The absence of the supraorbital foramen was previously mentioned in lions [13], tigers [30], dogs [29], sloth bears [31], and arctic foxes [32]. The lacrimal bone undistinguishable fused with the maxilla has been described in modern pangolins [33]. The unification of foramen rotundum and the orbital foramen to foramen orbitorotundum was reported in oxen and ruminants [34] and lions [13]. Above-mentioned features could be useful for identification of partially preserved skull fragments is archeological or forensic studies.

The massive rostrum with rostrally projected incisive bones has been described in subterranean rodents to serve as a support for nose pad, which is also prominent in hedgehogs and may be helpful in burrowing[35].

The specific strucuture of the tympanic bulla area—the loose ectotympanic connected by ligaments to the tympanic wing of the basisphenoid was one of the features described in other species as ancestral features of the therian mammals [36]. Due to the tympanic process of the petrosal part contributing to the bulla and basisphenoid forming its’ major part in erinaceids, Novacek includes this type of bulla to basisphenoid-petrosal type of the bulla [37, 38].

The anatomy of the parts that build up the tympanic bulla is similar to this described in the four-toed hedgehog *(Atelerix albiventris)*, described by Heffner et al. [39].

The study has potential limitations. The anatomical literature lacks similar description of other member of the Eulipotyphla order. There are a few articles and books on anatomy of those species, but not many uses digital imaging tools, hence the comparative aspect of this study may be limited [23, 24]. Due to the small size of an animal, high-quality diagnostic imaging is limited.

## Conclusions

This is the first study describing the radiological features of the skull of this species of hedgehog. The described anatomical features coincide with the features found in other species belonging to the order Eulipotyphla. Three different methods of visualizing the skull of the European hedgehog were used and compared. The results can be used by veterinary clinicians who work with wildlife. The study's results may be used to comparative anatomical studies and in creating diagnostic protocols for veterinary clinicians working with wildlife.

## Data Availability

The data used to support the findings of this study are available from the corresponding author upon reasonable request. The publication was financed by the Polish Minister of Science and Higher Education as part of the Strategy of the Poznan University of Life Sciences for 2024–2026 in the field of improving scientific research and development work in priority research areas.

## Abbreviations

WRC: Wildlife Rehabilitation Center
L: Lateral
VD: Ventro-dorsal
DV: Dorso-ventral
NAV: *Nomina Anatomia Veterinaria*

## Data availability

The data used to support the findings of this study are available from the corresponding author upon reasonable request.
